# Genetic Causes of Inner Ear Anomalies: a Review from the Turkish Study Group for Inner Ear Anomalies

**DOI:** 10.4274/balkanmedj.galenos.2019.2019.4.66

**Published:** 2019-07-11

**Authors:** Emre Ocak, Duygu Duman, Mustafa Tekin

**Affiliations:** 1 Department of Otolaryngology, Ankara University School of Medicine, Ankara, Turkey; 2 Division of Genetics, Department of Pediatrics, Ankara University School of Medicine, Ankara, Turkey; 3 Department of Audiology, Ankara University Faculty of Health Sciences, Ankara, Turkey; 4 John P. Hussman Institute for Human Genomics, University of Miami Miller School of Medicine, Miami, USA; 5 Department of Otolaryngology, University of Miami Miller School of Medicine, Miami, USA; 6 Dr. John T. Macdonald Department of Human Genetics, University of Miami Miller School of Medicine, Miami, USA

**Keywords:** Anomaly, genetics, inner ear, syndrome

## Abstract

Inner ear anomalies diagnosed using a radiological study are detected in almost 30% of cases with congenital or prelingual-onset sensorineural hearing loss. Inner ear anomalies can be isolated or occur along with a part of a syndrome involving other systems. Although astonishing progress has been made in research aimed at revealing the genetic causes of hearing loss in the past few decades, only a few genes have been linked to inner ear anomalies. The aim of this review is to discuss the known genetic causes of inner ear anomalies. Identifying the genetic causes of inner ear anomalies is important for guiding clinical care that includes empowered reproductive decisions provided to the affected individuals. Furthermore, understanding the molecular underpinnings of the development of the inner ear in humans is important to develop novel treatment strategies for people with hearing loss.

Hearing loss (HL) is the most commonly diagnosed sensory disorder affecting 1-2 of every 1000 newborns (1). Previous reports suggest that more than half of sensorineural HL (SNHL) is caused by genetic factors (2). Although the majority of cases with SNHL do not have additional findings, more than 400 genetic syndromes have been described as having SNHL as an accompanying feature.

Genetic or environmental causes disrupting the development of the inner ear (IE) lead to IE anomalies (IEAs) associated with SNHL. Bamiou et al. (3) reported that IEAs can be found in up to one-third of children with SNHL through a computerized tomography or a magnetic resonance imaging study.

Early diagnosis and timely management of SNHL is important as early intervention will have a large impact on the treatment outcomes. Understanding the genetic basis of SNHL associated with IEAs is important. This will help in making appropriate treatment decisions (e.g., feasibility of a cochlear implant), diagnosing other family members early, and empowering the affected individuals with family planning. The diagnosis may also be a sign of a syndrome involving other organ systems, which would necessitate screening for additional abnormalities.

Although significant progress has recently been made in understanding the physiology of hearing and pathologies leading to SNHL in humans, our understanding of the molecular mechanisms underlying the human IE development is astonishingly limited. Excellent studies have been performed in various animal models, particularly in zebrafish, chicken, and mouse, shedding light on the fundamental mechanisms involved in the vertebrate IE development (4,5,6). However, mutations in only a few of the genes identified in animal model systems have been shown to cause IEAs in humans, suggesting that the molecular mechanisms regulating IE development are only partially shared between different species.

Identification of the genetic basis of IEAs will provide much needed information about the development of the human IE, which could form the basis for novel therapies in the future. This review aims to summarize the current understanding of the genetic etiology of IEAs, specifically its clinical presentation, the genes involved, and the roles they play in embryology.

## MOLECULAR DEVELOPMENT of the INNER EAR

Most of the current knowledge about the molecular basis of IE development has been gained by studying model animals, specifically mouse, chicken, and zebrafish. There are general and organism-specific writing formats for genes and proteins. In general, symbols for genes are italicized, whereas symbols for proteins are not italicized. Human genes and proteins are written entirely in upper case, the first letter is capitalized for mouse genes and proteins, and for zebrafish, they are written entirely in lower case.

**Figure 1:**
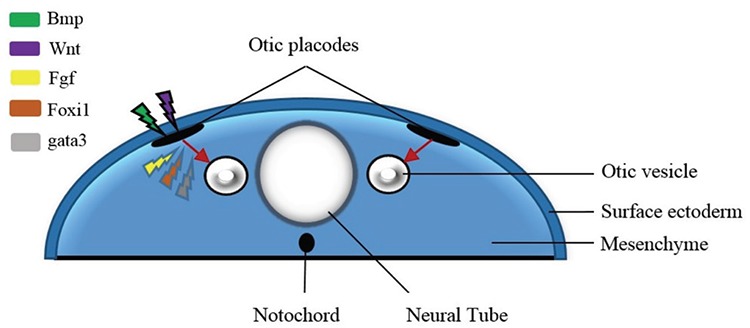
Differentiation of the otic placode in the preplacodal region and formation of the otic vesicle. The various molecules affecting the different stages of the process are shown in the figure.

Thickening of the ectoderm, which is known as the otic placode, constitutes the embryological basis of the IE. The development of the IE begins in the preplacodal region, which is a primitive embryological region that surrounds the neural plate to form the otic placode. Then, the otic vesicle forms from this thickened epithelium (Figure 1). This highly complicated process includes modeling the structure with morphogenetic movements, expression of cell-specific proteins, and differentiation of the highly specialized cell types. For later development of IE, planar polarization is necessary because stereociliary bundles are sensitive to vibrations only on a single plane, and it is also required for convergent extension, a polarized cellular movement that occurs during neural tube closure and cochlear extension (4). Several genes and numerous pathways work during these processes. Another issue on this subject is the influence of environmental factors on the molecular development of the IE structures. Epigenetic modifications and mtDNA play an important role in the molecular development of the IE (7).

### *Preplacodal region*

Studies have demonstrated that the interaction of the proteins in the fibroblast growth factor pathway, bone morphogenic protein (bmp), Wnt, and their antagonists play a major role in the development of the preplacodal region in the ectoderm of the developing embryo (8).

Gbx2 and Otx2 mutually modulate the anterior–posterior differentiation of the preplacodal region. Among them, Gbx is especially important in the development of the otic placode (9). The transcriptional coactivator Eya1 and the homeobox gene Six1 are believed to participate in the early differentiation of hair cells and neurons (10), which are in turn regulated by Foxi1 (11). Eya1 and Six1 together with Dach form a regulatory network that leads to transcriptional activation, cell proliferation, and organogenesis of the IE (12).

### *Otic vesicle*

The otic vesicle is formed from the invagination of the otic placode. In a study investigating the conditions that influence the response to Fgf during otic placode induction (13), signaling of this factor has been shown to induce the expression of zebrafish otic genes such as *pax8, pax2a, fgf24*, and *sox3* throughout the preplacodal region, which is important in setting the pattern of the otic vesicle. This indicates that the fibroblast growth factor pathway continues to be important in the induction of the otic placode. The abovementioned transformation is believed to be regulated by the *Hoxa1* gene (14). This pathway is also influenced by foxi1 (inhibition effect) and gata3 (promotion effect) during otic development (15). Sai et al. (16) investigated the RhoA activity for apical constriction during phase 2 IE placode invagination and found that invagination of the otic placode to form the otic vesicle occurs via the activation of myosin-II through not only fibroblast growth factor signaling but also the RhoA-ROCK pathway. After the development of the otic vesicle, the otocyst gives rise to the mature IE structures, including the vestibular system in the dorsal plane and the auditory system in the ventral plane.

### Molecules and factors involved in the neurogenesis of the inner ear

Several factors play important roles in the subsequent development of the neural IE structures after the otic vesicle stage. Inhibition of Sox2 transcription has been shown to be required for the progression of neurogenesis in the IE (17). In order for neuronal precursors to commit to a neuronal fate, Fgf10 signals the expression of Ngn1 and Neurod1, which act to inhibit the activity of Sox2 (17,18). Notch signaling also plays a role in specifying the sensory domains within the otic placode by inducing the proliferation of undifferentiated presensory cells, upregulating Sox2 activity, and inhibiting Ngn1 activity (19). Furthermore, tfap2a is believed to modulate the activity of fibroblast growth factor and notch by activating the inhibitor bmp7a, while playing a key role in neural development as well (20). Inner radial bundle formation is then mediated by Eph/Ephrin signaling, which is a target of Pou3f4 transcription factor activity (21,22).

### *Cochlea formation*

In the topographic anatomy of the temporal bone, the cochlea is located in the ventral plane in the anteroposterior development axis after the otic vesicle stage. Several factors and molecules are involved in the development of the cochlea such as *Jag1, Sox2*, and *Lfng* (23). Deletion of p27^kip1^ can cause some changes in the cochlea, such as overproduction of hair cells, interestingly causing SNHL (24). *Atoh1* is the earliest discovered factor expressed in the prosensory domain associated with sensory hair cells. In the early period, it can be detected in the entire cochlea; however, over time, the expression of *Atoh1* is restricted to hair cell progenitors. Numerous factors that can upregulate or downregulate *Atoh1* have been described. Among them, *Sox2* is one of the most investigated molecules. Although being required for the expression, *Sox2* also downregulates *Atoh1*. Other regulators of this transcription factor are the Id (inhibitor of differentiation) genes (*Id1, Id2, and Id3*). These genes are known to negatively regulate *Atoh1* (25). Cochlear lumen formation begins at the base of the cochlea and proceeds toward the apex. This is partially controlled by fluid secretion in the vestibular labyrinth, which is then absorbed into the endolymphatic sac, a process mediated by Slc26a4-encoded channels (26).

### *Semicircular canal development*

The vestibular system is located in the dorsal plane in the IE. The semicircular canals and their neural elements are derived from the two prominences of the otocyst, the horizontal and vertical canal pouches. The hindbrain is the source of the ventral–dorsal axial signaling for the IE. Wnts, from the dorsal hindbrain, are important signals for the development of the semicircular canals (27). Dlx5 has been shown to be one of the downstream genes that respond to Wnt signaling. Previous studies have demonstrated that the lack of Dlx5 can affect canal and crista formation (28). Another molecule that is also required for appropriate semicircular canal formation is Hmx3. On the other hand, *Chd7* encodes a chromodomain-containing helicase protein and has been proposed to act as a selector gene that encodes transcription factors essential for the formation of semicircular canals (29).

## CLASSIFICATION of INNER EAR ANOMALIES

IE malformations will be represented here according to the Sennaroglu classification system as it is one of the most widely used and accepted classification systems (30). From a systematic perspective, these malformations can be classified into eight distinct types as follows:

- Complete labyrinthine aplasia: This anomaly is also known as Michel deformity. There is an absence of the cochlea, vestibule, semicircular canals, and vestibular and cochlear aqueducts. There are three subgroups according to the imaging findings.

- Rudimentary otocysts: These are millimetric representations of the otic capsule without an internal acoustic canal. This type of anomaly represents between a complete labyrinthine aplasia and the common cavity.

- Cochlear aplasia: The cochlea is completely absent. The components of the vestibular system are in their normal anatomic locations.

- Common cavity: There is a cystic cavity representing the cochlea and the vestibule, but without showing any differentiation into the cochlea and the vestibule.

- Cochlear hypoplasia: Dimensions of the cochlea are smaller than normal. There is a differentiation between the cochlea and the vestibule with various internal architecture deformities. There are four distinct types of cochlear hypoplasia.

- Incomplete partition: This type of anomaly represents the most common IE malformation. There is a differentiation between the cochlea and the vestibule. The external dimensions are normal with various internal architecture defects. This group of malformations is subclassified into three subgroups as IP-I, -II, and -III.

- Enlarged vestibular aqueduct: The vestibular aqueduct is enlarged in the presence of a normal cochlea, vestibule, and semicircular canals.

- Cochlear aperture abnormalities: The cochlear aperture can be hypoplastic or aplastic, which can be detected in computerized tomography scans. This malformation may be accompanied by a narrow internal acoustic canal.

### Syndromic causes of inner ear anomalies in humans

IEAs can be sporadic or occur along with a part of a syndrome involving other systems. Numerous syndromes can be associated with IEAs in humans. The most frequently diagnosed ones with clinical findings and causative genes are summarized in Table 1.

### Nonsyndromic causes of inner ear anomalies

Nonsyndromic deafness is a type of hearing impairment in which HL is the only clinical finding in the patient. In these cases, the underlying etiology is generally a single gene mutation. Among all patients with nonsyndromic deafness, only a few genes have been discovered to cause IEAs when they have mutations.

### *SLC26A4*

In 1997, the gene responsible for Pendred syndrome was identified as *SLC26A4* (31). *SLC26A4* encodes a transmembrane protein termed as pendrin. Subsequently, *SLC26A4* mutations were also discovered in individuals with autosomal recessive nonsyndromic deafness associated with enlargement of the vestibular aqueduct (32). More than 200 mutations have so far been reported, which can be related to sporadic and familial forms of Pendred syndrome and nonsyndromic SNHL with enlargement of the vestibular aqueduct.

Recent studies applying molecular testing for *SLC26A4* mutations and radiological imaging of temporal bones have demonstrated that enlargement of the vestibular aqueduct can be recognized as the most penetrant feature of Pendred syndrome (33). Enlargement of the vestibular aqueduct is the most common radiological anomaly of the IE that is primarily identified in either of two different contexts, nonsyndromic enlargement of the vestibular aqueduct or Pendred syndrome. Although the mutant alleles of *SLC26A4* have been shown to correlate with the auditory and thyroid phenotypes, no connections between the type of mutation and the thyroid phenotype have been reported (34). Mutation in the *SLC26A4* gene is one of the leading causes of nonsyndromic SNHL and is inherited in an autosomal recessive manner.

Although variants in *FOXI1* and *KCNJ10* have been reported to cause SNHL with enlargement of the vestibular aqueduct when the same person is heterozygous for an *SLC26A4* mutation (i.e., digenic inheritance), this observation has not yet been confirmed by subsequent reports (35).

### *POU3F4*

Variants in the *POU3F4* gene at the locus DFN3 are a major cause of X-linked deafness worldwide. The X-linked *POU3F4* gene is the first nuclear gene implicated in nonsyndromic deafness. The type of HL may be SNHL or mixed and associated with IP-III (incomplete partition type 3), cochlear hypoplasia, and/or stapes fixation (DFN3) (36,37,38,39). In addition to mutations located within the gene, copy number variants not involving the coding part of the gene have been reported; de Kok et al. (40) identified a hot spot for microdeletions in patients with X-linked deafness 900 kb proximal to the DFN3 gene.

From a clinical perspective, when *POU3F4* is identified as the HL-causing gene, the surgeon should be alert for a perilymphatic gusher during stapes surgery. Another important issue to consider is that if the HL is found to be X-linked and associated with an IE malformation, mutations in *POU3F4* gene should be evaluated.

### *COCH*

The *COCH* gene is located at 14q11.2-q13 and encodes a secretory protein known as Cochlin (41). The pathogenetic mechanism of *COCH* gene-related HL is the accumulation of acidophilic deposits in the area of the spiral osseous lamina, spiral ligament, and vestibular nerve channels (42). Several reports indicate a high probability of a possible link between the mutations in the *COCH* gene and the presentation of IEAs. Hildebrand et al. (43) described a patient who presented with semicircular canal dehiscence associated with a mutation in the *COCH* gene. Dodson et al. (44) described a patient heterozygous for a mutation in the *COCH* gene and who showed an enlargement of the vestibular aqueduct upon computerized tomography imaging. Finally, de Varebeke described nine patients with the same mutation in the *COCH* gene. On computerized tomography imaging, eight of them were found to have sclerotic lesions and/or narrowing of the semicircular canals, and in one patient, the posterior vestibule was also affected (45). Based on these findings, *COCH* mutations are a possible autosomal dominant inherited cause of IEAs and have been postulated to play a role, along with type II collagen bundles, in laying down the structure of the IE (46).

### *ROR1*

*ROR1* is an integral transmembrane protein consisting of extracellular and intracellular conserved domains. There is one reported family with two children having congenital autosomal recessive nonsyndromic SNHL and a common cavity anomaly who had an *ROR1* gene mutation as the cause (47).

### *FOXF2*

Fox transcription factors regulate diverse biological processes throughout development and adult life. Mutations in some Fox genes are associated with human diseases. In a very recent paper, our study group published a homozygous mutation in the *FOXF2* gene required for cochlear development. In that study, the *FOXF2* gene mutation was found to be responsible for the IP-I anomaly (48).

Embryological development of the IE is a complex process in which various molecules are involved at different stages. Till date, only a few genes have been found to cause IEAs when their functions are disrupted by mutations. As the link between genetics and IEAs has been well established, future studies will help increase the knowledge regarding the molecular biology of IEAs.

## Figures and Tables

**Table 1:**
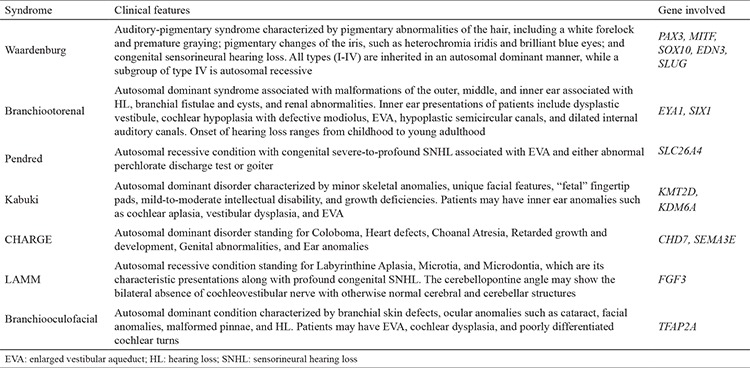
Syndromic causes of inner ear anomalies
